# Food Quantity Discrimination in Angelfish (*Pterophyllum scalare*): The Role of Number, Density, Size and Area Occupied by the Food Items

**DOI:** 10.3389/fnbeh.2020.00106

**Published:** 2020-06-23

**Authors:** Luis M. Gómez-Laplaza, Robert Gerlai

**Affiliations:** ^1^Department of Psychology, University of Oviedo, Oviedo, Spain; ^2^Department of Psychology, University of Toronto Mississauga, Mississauga, ON, Canada

**Keywords:** fish cognition, quantity discrimination, foraging, continuous variables, angelfish

## Abstract

Quantity discrimination, the ability to identify, process, and respond to differences in number, has been shown in a variety of animal species and may have fitness value. In fish, the ability to distinguish between numerically different shoals has been well studied. However, little work has been devoted to the investigation of such ability in a foraging context. Nevertheless, angelfish (*Pterophyllum scalare*) have been previously shown to be able to discriminate numerically different sets of food items, with variables such as size and density of the food items playing important roles in making the choice. Here, we examine the possible role of other numerical and non-numerical variables. Using a spontaneous binary choice task, we contrasted sets of food items differing in specifically controlled ways: (1) different numerical size but equal inter-item distance; (2) different numerical size and different inter-item distance; and (3) identical total contour length and area occupied but different individual food size and inter-food distance between the contrasted food sets. In Experiment 1, angelfish were found to prefer the sets with a large number of food items. In Experiment 2, they preferred the numerically smaller sets with clustered items to the numerically larger sets with scattered items, but only when the sets were in the large number range (10 vs. 5 food items). Finally, in Experiment 3 fish preferred numerically smaller sets with large-sized and scattered food items in the large number range sets. We conclude that food item number, density, and size may not be considered individually by angelfish, but instead, the fish respond to all these factors attempting to maximize energy gained from eating the food while minimizing energy expenditure collecting and/or protecting the food.

## Introduction

The ability to appraise the number of elements in a set of items or subjects may be critical when making decisions under any circumstances or in a variety of ecological contexts, including in intergroup conflicts (Benson-Amram et al., 2011; Bonanni et al., 2011), antipredatory responses (Wheatcroft and Price, 2018; Coomes et al., 2019), selecting territory (Nelson and Jackson, 2012), or in foraging (Panteleeva et al., 2013; Bogale et al., 2014). In all of these situations, numerical or quantity estimation ability may confer selective advantage (Nieder, 2017). Not surprisingly, such ability has been observed across a wide range of animal species. These include a variety of mammalian (Hanus and Call, 2007; Evans et al., 2009; Baker et al., 2012) and avian species (Rugani et al., 2013; Bogale et al., 2014; Tornick et al., 2015), but also reptiles (Gazzola et al., 2018; Miletto Petrazzini et al., 2018), amphibians (Krusche et al., 2010; Stancher et al., 2015), and invertebrates (Yang and Chiao, 2016; Gatto and Carlesso, 2019; see also Giurfa, 2019).

Fish, teleosts, and even sharks (Vila Pouca et al., 2019), have also been shown to possess the cognitive ability to distinguish numerically different sets of items. In the laboratory, most studies with fish have used spontaneous binary choice tests in a social context, in which experimental fish are faced with groups of conspecifics of different numerical size in a novel tank. Results generally show a preference by the test fish for the larger group (shoal), presumably because a greater number of shoal members represent a relative increase in protection against danger such as predators (Hager and Helfman, 1991). This choice has been experimentally shown in a large variety of fish species, but most work has been done in mosquitofish, *Gambusia holbrooki* (Agrillo et al., 2007, 2008, 2011), three-spined sticklebacks, *Gasterosteus aculeatus* (Frommen et al., 2009; Thünken et al., 2014; Mehlis et al., 2015), guppies, *Poecilia reticulata* (Piffer et al., 2012; Miletto Petrazzini and Agrillo, 2016; Lucon-Xiccato et al., 2017), zebrafish, *Danio rerio* (Pritchard et al., 2001; Potrich et al., 2015; Seguin and Gerlai, 2017), and angelfish, *Pterophyllum scalare* (Gómez-Laplaza and Gerlai, 2011a,b, 2015).

Although the above studies have sometimes used variations in the procedure and methodological approaches (reviewed in Agrillo and Bisazza, 2014; Agrillo et al., 2017), the choice-performance similarity across fish species suggests that the decision-making process may be the result of similar selection pressures and/or similar underlying cognitive mechanisms that process quantity related information. As mentioned above, comparable ability to choose between quantities that differ in the number of elements has been shown in other species across the animal kingdom. Similarities across multiple species in numerical abilities including quantity estimation may be interpreted as resulting from underlying evolutionarily conserved cognitive capacity (Feigenson et al., 2004). Alternatively, such similarities may also reflect independent, convergent evolution, i.e., similar natural selection. Either way, fish may be excellent species with which such cognitive abilities are investigated because they are simpler, evolutionarily more ancient than other vertebrates, and thus may allow one to explore the foundation of more complex numerical abilities seen in higher-order vertebrates (Shettleworth, 2010). They may be appropriate model systems to understand the rudiments of non-symbolic numerical cognition and its mechanisms (Messina et al., 2020).

Foraging behavior provides a good context within which one may examine the ability of fish to discriminate between sets differing in number because of the obvious fitness component of this task. Given that foraging has crucial fitness value across all species, it also affords comparative analysis. Preference for more numerous food item sets has been demonstrated in a variety of animal species (but see Panteleeva et al., 2013), including non-human primates (Hauser et al., 2000; Beran, 2001; Lewis et al., 2005), canids (Baker et al., 2011; Miletto Petrazzini and Wynne, 2016) birds (Hunt et al., 2008; Bogale et al., 2014), reptiles (Gazzola et al., 2018), amphibians (Uller et al., 2003) and invertebrates (Rodríguez et al., 2015). However, studies on food quantity discrimination in fish have been scarce and have been conducted so far only with two species: guppies, *Poecilia reticulata* (Lucon-Xiccato et al., 2015; Lucon-Xiccato and Dadda, 2017) and angelfish, *Pterophyllum scalare* (Gómez-Laplaza et al., 2018, 2019; Gómez-Laplaza and Gerlai, 2020). This may be due to technical difficulties, including the question of how to deliver well localized and stationary food items in the water and how to avoid potential confounding effects of olfactory cues. The paucity of this type of study in fish represents an obstacle to an adequate investigation of both mechanistic and evolutionary questions.

According to the optimal foraging theory (Stephens and Krebs, 1986), an individual should exhibit the most economical foraging pattern, i.e., maximize food (energy) intake while minimizing costs. Following this, Lucon-Xiccato et al. (2015) and Lucon-Xiccato and Dadda (2017) found guppies to choose the larger number of food items in a set, but when item number and size were conflicted, the guppies preferred the larger item size to numerically larger but smaller sized items. In spontaneous choice tests, angelfish have been shown to prefer the numerically larger to the smaller food set as long as the items were sized identically (Gómez-Laplaza et al., 2018). In this latter study, the response, as in other animal species, was found to be ratio-dependent with a decrease in accuracy as the numerical ratio between the two quantities approached one (in agreement with Weber’s law). However, numerical attributes were not dissociated from quantitatively varying non-numerical features, such as item density, overall contour length, surface area, or the size of the area where the food items were presented, i.e., the convex hull covering the food items. The controversy on whether the discrimination is guided by numerical or non-numerical attributes of the stimuli is a topic widely discussed nowadays (see Leibovich et al., 2017). Thus, understanding the relative importance of continuous vs. numerical cues-based discrimination is one of the most important challenges currently facing research on numerical cognition. Nevertheless, in fish, except for the study by Lucon-Xiccato et al. (2015), little attention has been devoted to empirically assess the influence of non-numerical, i.e., continuous, features of the stimuli on food quantity discrimination.

We have started to address this issue, and have begun the investigation of what features (including number) of the contrasted item sets angelfish utilize. In a previous study, we have found angelfish to prefer larger-sized food items with a preference limit of about 1.5:1 (ratio between the contour lengths of the items), although the numerical information of the sets also affected the choice (Gómez-Laplaza et al., 2019). Furthermore, when the number and size of the food items in the contrasted sets were kept constant, we found angelfish to discriminate the item sets based upon food density (inter-food item distance), preferring dense sets to sparsely arranged sets both in the small (3 vs. 3 food items) and in the large number range (5 vs. 5 food items; Gómez-Laplaza and Gerlai, 2020).

In the present study, we attempt to dissociate these and other non-numerical features from numerical attributes in the contrasting sets to deepen our understanding of the cues that govern the choice angelfish make in food quantity discrimination. First (Experiment 1), we study the relevance of the number of items in a set when the density and size of the items (continuous variables) are maintained constant. Next (Experiment 2), we examine the influence of the number of items and density when these features are in conflict while keeping the size of the food items equal. Last, another non-numerical variable that has been shown to influence non-symbolic numerical discrimination is the convex hull, the perimeter that encompasses all of the items in a set (e.g., DeWind and Brannon, 2016; Gilmore et al., 2016; Braham et al., 2018). This variable usually covaries with other continuous as well as numerical attributes of contrasted sets in food quantity discrimination studies with fish. For example, in the preceding experiments, as in most food item number discrimination studies, more food items occupied more space, had larger cumulative contour length, and also had a larger convex hull. In Experiment 3, we dissociated the potential effect of the convex hull from those of other factors by keeping the convex hull of the food sets (as well as the overall contour length of the items) in the contrasted sets equal and varying the number and density of items.

## Materials and Methods

### Subjects and Housing Conditions

Juvenile individuals of the freshwater cichlid species, angelfish *Pterophyllum scalare*, obtained from local suppliers were used in the current experiments. Juveniles of 3.0–3.3 cm standard length were maintained in our laboratory in holding glass aquaria (60 × 30 × 40 cm, length × width × depth) for at least 2 weeks before the experimental procedures began. Juveniles of this size and age do not yet exhibit sexual or courtship behaviors and thus form sex-ratio independent uniform shoals in nature and the laboratory (Praetorius, 1932; White, 1975). Each aquarium was provided with a gravel substratum and dechlorinated tap water, continuously cleaned by external filters. The water temperature was kept at 26 ± 1°C using thermostat-controlled heaters. Each aquarium was illuminated by a 15-W white fluorescent light tube (12:12 light:dark cycle) and was lined on the outside with white cardboard, except the front to allow observation by the experimenter. The fish were fed commercial flake food delivered to the water surface twice daily: at 10:00 h and 18:00 h.

### Experimental Apparatus

**Figure 1:**
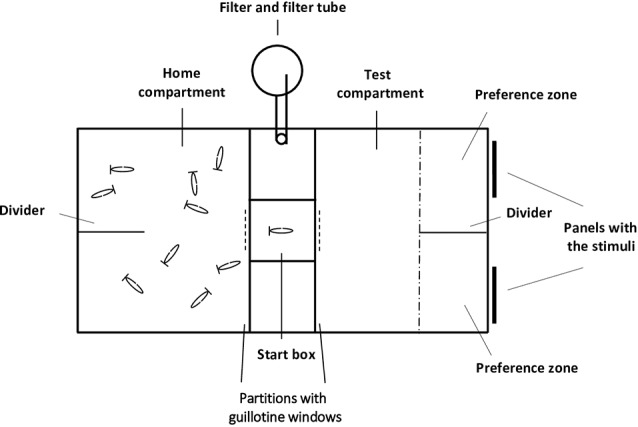
Schematic top view representation of the experimental aquarium. The home and test compartments were separated by a smaller middle compartment (10 × 30 × 33 cm, length × width × depth) whose central part constituted the start box (10 × 10 × 33 cm length × width × depth). Partitions, with guillotine windows, delimited the compartments; panels (placed outside the experimental aquarium) where the stimuli were presented; and a transparent plastic divider that delimited the two preference zones (10.5 × 15 cm length × width) are indicated. Panels, also with guillotine windows (dashed line), were superimposed on the partitions when tests began, the experimenter could raise or lower these panels to open or close the guillotine windows. The guillotine window of the transparent partition delimiting the testing compartment was 14.5 cm away from but in front of, the transparent divider. The position of the home compartment and the testing compartment was counterbalanced to prevent any lateral side bias.

The experimental aquarium used (60 × 30 × 33 cm, length × width × depth) was the same we described in recent studies (Gómez-Laplaza et al., 2018, 2019), and was kept under the same conditions (substratum, temperature, light, and photoperiod) as the holding aquaria. All walls of the aquarium were lined with non-transparent white cardboard on the outside to prevent the potential influence of external visual stimuli, and fish were recorded with a video camera from above. Two transparent partitions (placed 25 cm from each lateral short side) divided the aquarium into three compartments. Each partition had a small rectangular guillotine window in the center that could be opened (and closed) by the experimenter to allow fish to pass through (or not) between compartments (Figure 1).

The two lateral compartments were considered the “home compartment” and the “test compartment” and their role was interchanged across fish. The central part the start box from where the experimental fish were released at the start of the behavioral tests. A transparent plastic divider placed in the middle of each of the lateral compartments delimited two equally-sized halves: the “preference zones” where the stimuli were simultaneously presented during testing. To avoid diffusion of potential chemical cues, the food stimuli were presented outside the experimental aquarium and were pasted on 7 cm × 7 cm area (positioned 7 cm from the bottom of the aquarium) at the terminal part of transparent plastic panels. Thus, only visual cues of the food items were accessible for the test fish, and the panels with the food items were positioned flush against the exterior sidewall of the testing compartment. The distance between the two panels was 10 cm, and the panels were inserted between the glass and the white cardboard lining the wall. The food sets remained visible during the test period and could not be eaten by the experimental fish.

Discrete food items were prepared by making a homogeneous mass with aquarium fish flakes using some water. The mass was agglutinated, and circular pieces of different sizes, depending on the experiment, were obtained using a methacrylate mold sheet (1 mm thick). This was perforated with holes of different diameters into which portions of the agglutinate were introduced to obtain food items with the size required.

### Procedure

A detailed description of the recently developed procedure can be found in Gómez-Laplaza et al. (2018; 2019; see also Figure 1). This experimental procedure was developed to minimize the handling of experimental fish and reduce isolation-induced stress. Importantly, experimental fish in this procedure were allowed to voluntarily enter the start box and this entry marked the start of testing. The procedure consisted of two phases. During the habituation phase, shoals of 10 angelfish were placed into the experimental aquarium, and for 7 days they could explore the three compartments and pass through the guillotine windows freely. During this phase, discrete food items of several sizes were pasted onto the lower part of transparent panels that were lowered into the water during each feeding session, to habituate the fish to this type of food delivery. To reduce potential monopolization of food, panels with the food were distributed along the long walls of the experimental aquarium, and variable distribution of the food items prevented angelfish from associating the food with one specific location in the aquarium. The feeding schedule and amount of food available remained as in the holding aquaria. The second phase of the experiment was the test phase. During this phase, subjects continued to stay with their shoal mates, and a subject was tested only when it voluntarily entered the start box from the home compartment on the way to the test compartment, where two sets of food items were presented (Figure 1).

Before starting each trial, an opaque white partition identical to the transparent partitions, including the guillotine window, was superimposed over one of the transparent partitions, covering it. All 10 experimental fish were kept in the compartment now delimited by the opaque white partition (home compartment), and the opaque guillotine window was closed, thus blocking the view of the other side of the aquarium. The other guillotine window in the transparent partition that delimited the other compartment (the test compartment) was also closed, in this case with a transparent panel. Subsequently, the corresponding sets of food items were placed on the external side of each of the preference zones of the test compartment. After a 3-min period, the experimenter raised the opaque white guillotine window of the home compartment to allow fish to spontaneously pass through it into the start box. The window was immediately closed, and the remaining subjects could not see what was happening on the other side of the partition. The subject remained in the start box for 30 s during which it could see the two sets of food items through the transparent partition. Afterward, the transparent guillotine window was raised to allow the fish to freely enter the test compartment to make the choice.

Tests took place in the morning at the usual feeding time (10:00 h–10:15 h), thus subjects were not food-deprived, but they were sufficiently motivated to perform the task. The behavior and position of the subjects were recorded for 5 min. This recording period was chosen to enhance the replicability of findings, i.e., to make our procedure compatible with that of previous studies in which the same period of testing was employed (e.g., Gómez-Laplaza et al., 2018, 2019). We also counterbalanced the left-right presentations of the stimuli across fish to control for possible side preferences. We also pseudo-randomized the order of presentation of each stimulus combination across subjects.

**Figure 2:**
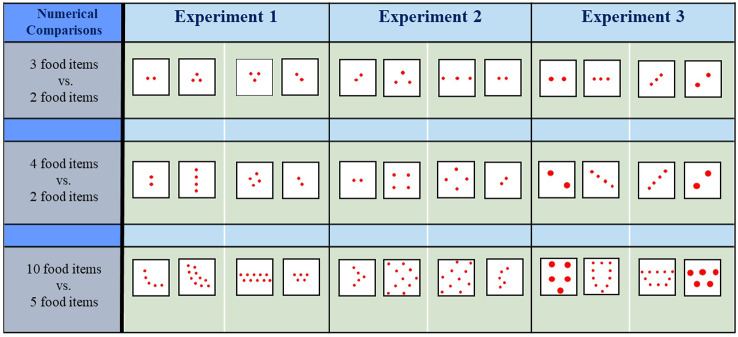
Examples of the contrasting food sets tested in each of the experiments and the corresponding numerical comparisons. Drawings are not at scale.

In each experiment, 12 different angelfish were tested in each of the three contrasts presented (i.e., a total of 36 fish). We performed three experiments, thus a total of 108 fish were used in this study. To avoid potential bias arising from a putative species-specific configuration pattern preference, a different configuration of the items was presented to each fish in each of the contrasts of each experiment, i.e., 12 different arrangements of the items in each contrast were employed (also see Figure 2). Consequently, each fish was tested only once, i.e., each fish saw a single pair of contrasts.

After having been tested, each subject was removed from the experimental aquarium and placed in another tank where it was fed. Likewise, the rest of the fish of the shoal was fed in the usual way in the experimental aquarium, after raising the guillotine windows and removing the stimulus sets. Every second day, after two fish had been tested and removed, two new fish were transferred to the experimental aquarium to maintain the size of the shoal in the experimental aquarium relatively constant. At the end of the study, all fish were returned to the supplier.

### Recording and Statistical Analysis

We recorded the first choice made by the experimental angelfish (i.e., the first preference zone close to the stimuli the fish entered after leaving the start box). Also, we recorded the frequency of entries (i.e., the number of times fish entered each of the preference zones), the latency (sec) to enter the preference zones (i.e., the time elapsed since the experimental fish left the start box and enter each preference zone), and the time spent (s) in each preference zone. This time was considered a measure of the test fish’s preference for the corresponding set. For each fish, an index to quantify preference for one set over the other was calculated as the proportion of time spent in the preference zone near the larger set relative to the total time spent in both preference zones.

In each experiment, tests for normality (Shapiro–Wilk test) and equality of variance (Levene’s test) were performed on the data before analysis. If the data were not normally distributed, they were log-transformed before analysis to meet the assumptions of parametric statistics. This transformation was performed in the latency data of all experiments. A one-sample *t*-test was used to investigate whether the observed preference index was significantly (*p* ≤ 0.05) different from chance (preference index value at chance = 0.5). The Holm-Bonferroni sequential correction method was used to correct for type I error resulting from multiple comparisons. The effect size for significant results was calculated by using Cohen’s *d*. A Cohen’s *d* of about 0.2, 0.5, and 0.8 represents a small, medium, and large effect size, respectively. One-way ANOVA for independent samples was used to analyze the effect of the contrasts on preference. In case of a significant effect, it was followed by Tukey Honestly Significant Difference (HSD) *post hoc* multiple comparison test. The effect size for significant results was calculated using partial eta-squared ($\eta_{\text{p}}^{2}$). Binomial probability tests comparing the number of fish initially choosing one set or the other were used for each pair of contrasting sets, and frequency and latency scores were analyzed using paired *t*-tests (see Table 1). All tests employed were two-tailed. All subjects entered both preference zones at least once.

**Table 1:** Performance of angelfish (*N* = 12 in each comparison) when faced with the different numerical contrasts.

Contrasts	First choice (out of 12 fish)^a^			Frequency of entries^b^				Latency to enter^c^			
	Smaller food set	Larger food set	Binomial test	Smaller food set	Larger food set	*t*-test		Smaller food set	Larger food set	*t*-test	
						*t*_(11)_-value	Probability			*t*_(11)_-value	Probability
Experiment 1 (equal density)										
3 vs. 2	1	11	*P* = **0.006**	3.58 ± 0.45	3.67 ± 0.40	0.209	*P* = 0.838	48.58 ± 9.47	7.42 ± 5.43	5.518	*P* < **0.001**
4 vs. 2	1	11	*P* = **0.006**	2.42 ± 0.26	4.00 ± 0.48	2.656	*P* = **0.022**	76.92 ± 14.99	12.67 ± 8.16	3.978	*P* = **0.002**
10 vs. 5	2	10	*P* = **0.039**	3.08 ± 0.36	4.42 ± 0.56	1.750	*P* = 0.108	64.17 ± 15.51	21.58 ± 16.47	3.077	*P* = **0.011**
Experiment 2 (different density)										
3S vs. 2C	8	4	*P* = 0.388	3.75 ± 0.51	2.50 ± 0.50	1.546	*P* = 0.150	32.75 ± 11.32	55.08 ± 14.12	1.077	*P* = 0.304
4S vs. 2C	8	4	*P* = 0.388	6.25 ± 0.72	6.83 ± 1.07	0.754	*P* = 0.466	6.67 ± 2.33	21.42 ± 5.98	1.624	*P* = 0.133
10S vs. 5C	8	4	*P* = 0.388	5.08 ± 0.38	3.67 ± 0.41	2.376	*P* = **0.037**	16.08 ± 6.74	30.17 ± 12.03	1.606	*P* = 0.137
Experiment 3 (equal convex hull)										
3CS vs. 2SL	10	2	*P* = **0.039**	5.00 ± 0.56	3.42 ± 0.31	2.777	*P* = **0.018**	10.17 ± 6.30	45.42 ± 11.42	3.073	*P* = **0.011**
4CS vs. 2SL	8	4	*P* = 0.388	4.42 ± 0.47	3.17 ± 0.39	1.820	*P* = 0.096	17.33 ± 8.30	45.17 ± 14.07	1.482	*P* = 0.166
10CS vs. 5SL	9	3	*P* = 0.146	5.67 ± 0.70	3.58 ± 0.45	2.661	*P* = **0.022**	27.58 ± 17.78	66.58 ± 18.68	1.530	*P* = 0.154

*Experiment 1: different number of food items, but equal density in the sets; Experiment 2: food items clustered in the smaller sets and scattered in the large sets; Experiment 3: food sets with equal convex hull and contour length, consequently food items had different density and size. Note. Subjects were tested individually. Descriptive statistics include means ± SE. The tests used to compare the scores are also included. ^a^Number of fish whose first choice was one or the other stimulus set. ^b^Frequency, number of times that subjects entered to the preference zones. ^c^Latency to enter the preference zone near one or the other stimulus set. Arrangement of the food sets: C, Clustered; S, Scattered; SL, Scattered and Large items; CS, Clustered and Small items. Statistically significant effects are shown in bold*.

### Experiment 1: Contrasting Sets With Different Number of Same-Sized Food Items and Equal Inter-Item Distance

#### Methods

A previous study with angelfish showed that dense, clustered sets (with inter-food item distance of 4 mm) are preferred over scattered sets (with inter-food item distance of 8 mm) when the number of food items was equal in each set (Gómez-Laplaza and Gerlai, 2020). The preference was exhibited both when sets were constituted of a small number of food items (≤4 items) and a large number of food items (>4 items). However, no attempt was made to examine the influence of the density of the items when the number of items is different in each of the contrasting sets. In the present experiment, we try to fill this gap and extend the findings of our previous study by investigating the performance of angelfish in a choice task where the number of items in the contrasted sets differs while the density (inter-item distance) of the items remains equal. Since in the previous study the inter-food item distance to which fish was responsive was 4 mm, here the distance between any of two adjacent items was also kept at 4 mm, and the diameter (size) of each item was 2 mm. We tested quantities both in the small (3 vs. 2 food items, and 4 vs. 2 food items) and the large number range (10 vs. 5 food items; Figure 2). The contrast 4 vs. 3 was not tested since angelfish were previously found not to exhibit a preference for any of the sets in this comparison (Gómez-Laplaza et al., 2018). We hypothesized that if angelfish can discriminate between the different quantities, they should choose the set with the larger number of food items as it would maximize food intake. However, when the quantities of contrasted food items are large in both sets, the adaptive significance of preferring a larger number of items may diminish given that even the smaller set would satiate the fish (e.g., Gill, 2003). The 10 vs. 5 contrast may represent this situation. We also note that although the density and size of the items were maintained equal in the sets, the preference for the numerically larger sets does not necessarily indicate that individuals rely exclusively on the number of items since non-numerical variables that covary with numbers, such as the convex hull and overall contour length of the items, could also influence the choice.

#### Results

**Figure 3:**
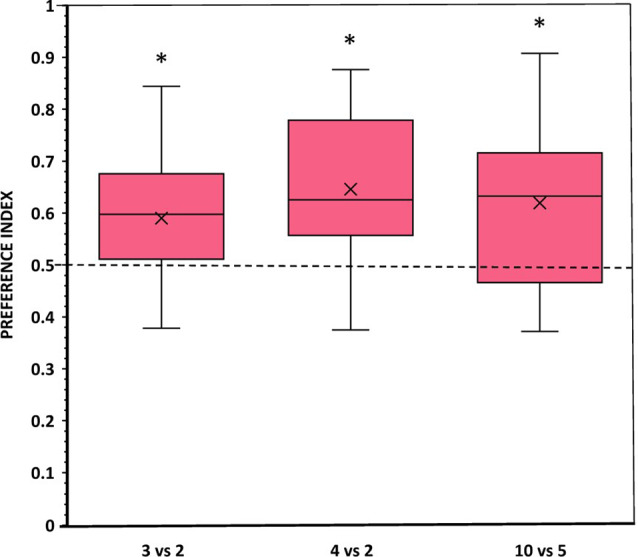
Choice of angelfish in the different numerical contrasts tested when the size and density (inter-item distance) of the items in each contrast were equal. The proportion of time (preference index) spent by test fish in the preference zone close to the food sets. Box plots show median (horizontal line in the boxes), 25% and 75% quartiles (boxes), and the highest and lowest values within the range of 1.5 times the respective quartiles (whiskers). Blades represent the mean proportion value. Values above 0.5 indicate a preference for the numerically larger food sets. A significant departure from the null hypothesis of no preference is indicated by asterisks, *p < 0.05.

When angelfish were presented with comparisons consisting of 3 vs. 2 and 4 vs. 2 food items (i.e., within the small number range) or with 10 vs. 5 food items (i.e., in the large number range), they spent significantly more time close to the set with the larger number of items compared to chance (*t*-test with Holm-Bonferroni correction: *t*_(11)_ = 2.350, *p* = 0.038, *d* = 0.68; *t*_(11)_ = 3.335, *p* = 0.021, *d* = 0.96; *t*_(11)_ = 2.631, *p* = 0.046, *d* = 0.76, respectively; Figure 3). Furthermore, in all comparisons, a significant number of fish (10–11 out of the 12 experimental fish), chose first the preference zone close to the numerically larger set (binomial test, all *p* ≤ 0.039; Table 1). In line with these findings, the latency to approach the larger food set was also significantly lower than the latency to approach the smaller set in all comparisons (all *p* ≤ 0.011, all *d* ≥ 1.68; Table 1). Notably, angelfish entered both preference zones several times in all contrasts, suggesting active exploration and implying a lack of fear/anxiety. Furthermore, the number of entries (frequency) appeared higher in the zone close to the larger food set compared to that in the smaller food set, a difference that was found significant only in the 4 vs. 2 contrast (*p* = 0.022, *d* = 1.21; Table 1).

One-way ANOVA showed that the difference in the magnitude of the response for the larger food set between the three contrasts was not significant (*F*_(2,33)_ = 0.427, *p* = 0.656), possibly indicating lack of difference in the strength of motivation to choose the larger number of food items among the contrasts.

### Experiment 2: Contrasting Sets With Different Number of Same-Sized Food Items and Different Inter-Item Distance

#### Methods

In Experiment 1, all food items had the same size and the distance between them was also kept constant. Under these circumstances, angelfish preferred the numerically larger sets to the smaller sets, a finding that supports prior results (Gómez-Laplaza et al., 2018). Previously, we have also shown that angelfish prefer dense (clustered) sets (inter-item distance: 4 mm) to scattered sets (inter-item distance: 8 mm) other features being equal (Gómez-Laplaza and Gerlai, 2020). In the second experiment, we conflicted with these two features, i.e., the number of the items and density of the items (inter-item distance). We presented contrasts 3 vs. 2, 4 vs. 2 and 10 vs. 5 food items (in all, the diameter of food items was 2 mm), but now in the smaller set the items were close to each other (clustered condition, 4 mm inter-item distance, the preferred distance found previously), and in the larger set the items were further apart (8 mm inter-item distance, the scattered condition; Figure 2, Experiment 2). The aim was to test which of the two features take priority in the preference. If angelfish prioritize number over density, they should choose the larger sets in the contrasts. However, if they prioritize density, they should choose the smaller sets with the clustered condition.

#### Results

**Figure 4:**
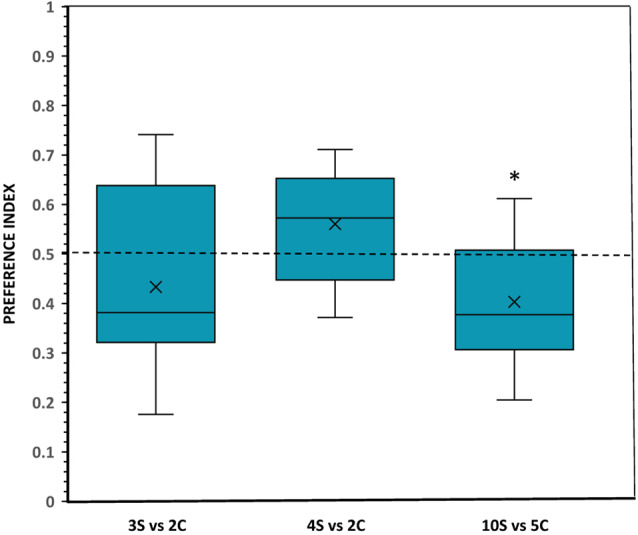
Choice of angelfish in the different numerical contrasts when the size of the items was equal but the density (inter-item distance) was different: clustered (C) and scattered (S). The proportion of time (preference index) spent by test fish in the preference zone close to the food sets. Box plots show median (horizontal line in the boxes), 25% and 75% quartiles (boxes), and the highest and lowest values within the range of 1.5 times the respective quartiles (whiskers). Blades represent the mean proportion value. Values above 0.5 indicate a preference for the numerically larger, scattered food sets, whereas values below 0.5 indicate a preference for the numerically smaller, clustered food sets. A significant departure from the null hypothesis of no preference is indicated by asterisks, *p < 0.05.

No significant departure from chance was found in the preference by the subjects when they were confronted with a choice between a set of three scattered food items and a set of two clustered food items. For example, the analysis of the amount of time the experimental fish spent in each zone of preference found no significant difference compared to chance (*t*_(11)_ = 1.240, *p* = 0.241; Figure 4). Latency to enter the preference zones (*t*_(11)_ = 1.077, *p* = 0.304), the frequency of entries to these zones (*t*_(11)_ = 1.546, *p* = 0.150) and the number of fish first choosing one or the other stimulus set all showed no significant effects (binomial, *p* = 0.388; Table 1).

A similar performance was found when fish had to choose between sets of 4 scattered food items vs. 2 clustered food items. In this comparison, angelfish showed a non-significant preference for spending more time near the numerically larger set with the scattered items (*t*_(11)_ = 1.877, *p* = 0.087; Figure 4). Likewise, all other behavioral parameters measured (first choice, latency to enter the preference zones, and frequency of entries) indicated the absence of preference for one over the other food set (all *p* > 0.05; Table 1). Interestingly, in both 3 vs. 2 and 4 vs. 2 contrasts angelfish showed a slight, but non-significant, tendency to prefer the smaller clustered sets, e.g., in the number of fish that first chose those sets and in the latency to enter the preference zone close to the smaller clustered sets (Table 1).

When the fish faced sets containing a number of food items within the large number range (10 scattered items vs. 5 clustered items), a preference significantly different from chance was shown for the numerically smaller but clustered set (*t*_(11)_ = 2.604, *p* = 0.025, *d* = 0.75; Figure 4). Furthermore, the number of entries to the preference zone near the smaller and more clustered set was significantly higher than the number of entries to the zone close to the larger, scattered set (*p* = 0.037; *d* = 0.96; Table 1). A similar, but non-significant, tendency to prefer the smaller and clustered set was found for latency to enter the preference zone close to that set and for the number of fish first choosing that set (Table 1).

One-way ANOVA revealed a significant difference between treatments in the magnitude of the response (*F*_(2,33)_ = 3.93, *p* = 0.030, $\eta_{\text{p}}^{2}$ = 0.19), and Tukey test revealed that this effect was mainly due to the difference in preference between the contrasts 4 scattered vs. 2 clustered and 10 scattered vs. 5 clustered (*p* = 0.033).

### Experiment 3: Contrasting Sets With a Different Number, Item Size and Inter-Item Distance, but Equal Convex Hull and Overall Contour Length

#### Methods

In the preceding experiments, same-sized food items were used in such a way that the sets with a larger number of items also had a greater amount of food. Notably, in the numerically larger set, the cumulative contour length of the food items, as well as the space occupied by the food items, was larger compared to corresponding parameters in the numerically smaller sets. In the above experiments, and also in our previous studies, convex hull (the minimum convex polygon containing all items) was not a continuous variable that played a critical role in food quantity discrimination, co-varying with number and density of the items in the sets. Also, when number and density were controlled by maintaining them identical and only the configuration was varied (and consequently the convex hull too), angelfish failed the discrimination (Gómez-Laplaza and Gerlai, 2020). The results indicated that the spatial configuration (and, thus, the convex hull) was not a prominent variable affecting the choice.

Likewise, the role played by the cumulative contour length of the items (the sum of the diameter of the circular food items) was not controlled in Experiments 1 and 2. However, this feature of the stimuli has been shown to influence food quantity discrimination in angelfish (Gómez-Laplaza et al., 2019). Also, the convex hull has been shown to affect performance in other animal species (Gatto and Carlesso, 2019). Despite these findings, neither of these non-numerical variables (contour length and convex hull) has received sufficient attention in food quantity discrimination studies conducted with fish in the past. Additionally, an important limitation attributed to spontaneous choice tests of quantity discrimination is that only one variable is usually controlled at a time. Considering the above, in the current experiment, we controlled two variables simultaneously: cumulative contour length and convex hull.

Notably, these two factors have been tested in our previous studies (Gómez-Laplaza et al., 2019; Gómez-Laplaza and Gerlai, 2020). Variation of the convex hull was found to have little effect on food quantity discrimination. On the contrary, the cumulative contour length of the items was found to affect discrimination. In the current experiment, both these features (convex hull and contour length) were maintained constant. That is, the experiment was designed to dissociate the potential effects of these two variables from those of numerosity. To this aim, we presented experimental angelfish with sets containing a different number of food items (3 vs. 2, 4 vs. 2, and 10 vs. 5), but now we equated both convex hull and total contour length between the contrasted sets by changing the size and the density of the items (Figure 2; Experiment 3). To maintain the cumulative contour length of the sets equal, the size of the food items in the numerically smaller sets needed to be larger than in the numerically larger sets. Therefore, in the 3 vs. 2 contrast, the diameter of the items in the set of 2 was 1.5 times greater (3 mm) than in the set of 3 (2 mm), thus equating for contour length. Also, the inter-item distance for the set with the two items was 8 mm whereas it was 4 mm for the three-item set. In the numerical contrast 4 vs. 2, each item of the two-item set had a 4 mm diameter and an inter-item distance of 12 mm, and each item of the four-item set had 2 mm diameter and an inter-item distance of 4 mm. In the contrast 10 vs. 5, each item of the five-item set had a 4 mm diameter and an inter-item distance ≥6 mm (depending on the configuration), whereas each item of the 10-item set had 2 mm diameter and an inter-item distance of 4 mm. Making the overall space occupied by the sets (convex hull) and the overall contour length of the items (approximately total amount of potential food) constant, we argued, would allow us to dissociate these non-numerical features from others upon which angelfish may base their discrimination of the sets, including numerical attributes. We emphasize that in this experiment the size of the items was larger in the numerically smaller sets, whereas the size of the items was smaller in the numerically larger sets. Under these circumstances, according to a previous study (Gómez-Laplaza et al., 2019), angelfish should favor the numerically smaller sets. On the other hand, in the numerically larger sets the items were closer to each other, a feature that was found to be preferred by angelfish before (Gómez-Laplaza and Gerlai, 2020). Thus, the choice made by angelfish will depend upon on how these fish weigh the importance of features such as item size, item distance, and item number, i.e., upon how these features interact and influence the fish’s decision making.

#### Results

**Figure 5:**
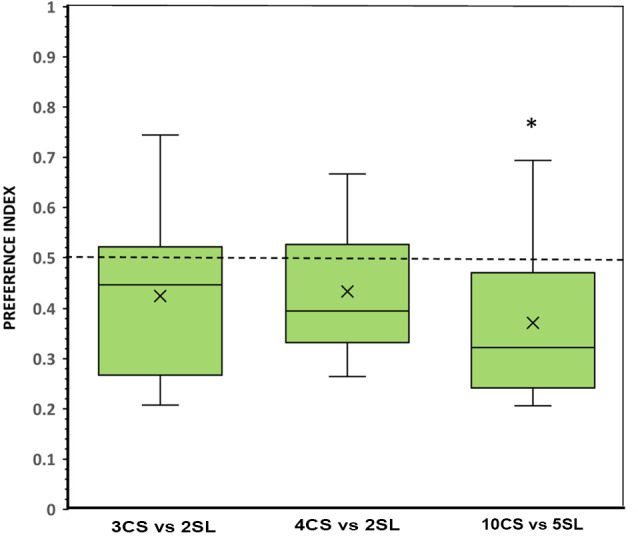
Choice of angelfish in the different numerical contrasts when the convex hull and contour length was equated between the contrasting sets, but the size and density of the items were different. The proportion of time (preference index) spent by test fish in the preference zone close to the food sets. Box plots show median (horizontal line in the boxes), 25% and 75% quartiles (boxes), and the highest and lowest values within the range of 1.5 times the respective quartiles (whiskers). Blades represent the mean proportion value. Values above 0.5 indicate a preference for the numerically larger sets with clustered and small items, whereas values below 0.5 indicate a preference for the numerically smaller sets, with scattered and large items. A significant departure from the null hypothesis of no preference is indicated by asterisks, *p < 0.05. SL, scattered and large food items; CS, clustered and small food items.

In the 3 vs. 2 discrimination test with the food items presented in the numerically smaller set being more scattered and size-wise larger than in the numerically larger set (with the convex hull and the contour length of the food items made equal between the two sets), subjects showed no significant difference compared to chance in the time spent in either preference zones (*t*_(11)_ = 1.643, *p* = 0.129; Figure 5). However, a preference for the numerically smaller set with larger and scattered items was revealed by the significantly higher number of fish that first chose that set (binomial, *p* = 0.039; Table 1), and by the shorter latency to enter (*p* = 0.011, *d* = 1.67) and a higher number of times that fish entered the zone close to that set (*p* = 0.018, *d* = 0.98; Table 1). For the 4 vs. 2 food item contrast, we also did not find a statistically significant departure from chance in the preference index (*t*_(11)_ = 1.768, *p* = 0.105; Figure 5). The other parameters also yielded results indicating no significant preference for any of the food sets contrasted (all *p* > 0.05; see Table 1). It is noteworthy that, in all parameters considered, subjects showed a tendency, albeit not significant, to prefer the smaller set with the larger and more scattered items.

A different performance was found when comparing sets in the large number range (i.e., 10 vs. 5 food items). Fish showed a preference significantly above chance for the location where the numerically smaller set, with larger and scattered food items, was presented (*t*_(11)_ = 2.785, *p* = 0.018, *d* = 0.80; Figure 5). This tendency of preference is supported by the analysis of the frequency of entries to the preference zones, which did find a significantly higher number of entries to the zone close to the numerically smaller set with size-wise larger and more scattered food items (*p* = 0.022, *d* = 1.01; Table 1). The latency to approach one or the other set and the number of fish that first chose one or the other set showed a similar tendency favoring the numerically smaller set with scattered items of larger contour length, although differences were not significant (see Table 1).

One-way ANOVA revealed that the difference in the magnitude of the preference for the numerically smaller set with larger and scattered food items was not significantly different between the three contrasts tested (*F*_(2,33)_ = 0.613, *p* = 0.548). When comparing the results (preference index) of each contrast in the three experiments (conditions), a significant difference among the performance of the subjects was found (one-way ANOVA, 3 vs. 2: *F*_(2,33)_ = 4.025, *p* = 0.027; 4 vs. 2: *F*_(2,33)_ = 7.962, *p* = 0.002; and 10 vs. 5: *F*_(2,33)_ = 9.796, *p* < 0.001), and Tukey *post hoc* test revealed that in all contrasts the results of Experiment 1 (equal density, and preference for the larger food set) were significantly different from those of Experiment 3 (equal convex hull and contour length of the sets, and a tendency to prefer the smaller food set with larger and scattered items; all *p* ≤ 0.043).

## Discussion

In this study, we attempted to further our knowledge on the potential role numerical and non-numerical factors may play in quantity discrimination in angelfish in the context of foraging. We found angelfish to be able to discriminate between food sets that differed by one, two, or five discrete food items. In all conditions, experimental angelfish preferred the more numerous set to the less numerous set when the inter-item distance was equated. These findings are consistent with, and also confirm and extend, those of a previous study with angelfish where the same and similar comparisons were tested but the inter-item distance (density) of the items in the sets was not controlled (Gómez-Laplaza et al., 2018). Overall, these findings indicate that angelfish can process quantitative information for obtaining food. Our results also align with those reported by Lucon-Xiccato et al. (2015), who found guppies to generally prefer the larger food set over the smaller one. Despite the disparity of the sets, the motivation of angelfish to choose the numerically larger set was apparently similar in the three conditions. The latency to enter the zone close to the numerically larger set, the number of fish that first chose that zone, and even the frequency of entries, indicating greater persistence for feeding in that zone, all demonstrated the preference for the set with a larger number of food items. Even in the 10 vs. 5 contrast, five items may represent a sufficient quantity to satiate an individual, nevertheless, angelfish still chose the larger number of food items. Such preference for the larger number of items is comparable to what has been found in numerous other studies (e.g., Hauser et al., 2000; Feigenson et al., 2002; Hanus and Call, 2007; Krusche et al., 2010; Garland et al., 2012; Stancher et al., 2015).

In a social context, angelfish have also shown discrimination abilities comparable to those found in the current study (Gómez-Laplaza and Gerlai, 2013). When angelfish were given a choice between two shoals of conspecifics, including 3 vs. 2 and 10 vs. 5 with inter-fish distance kept constant within each stimulus shoal, they spent significantly more time close to the larger shoal compared to chance (Gómez-Laplaza and Gerlai, 2013). Systematically varying the characteristics of the stimuli used, while employing the same or similar numerical contrasts as in the current study, preference by angelfish for the numerically larger stimulus shoal was generally found (Gómez-Laplaza and Gerlai, 2011a,b, 2015, Gómez-Laplaza and Gerlai, 2016a,b; 2017). Thus, our current results together with previous ones indicate the robustness of the quantity discrimination abilities of angelfish across two distinct contexts, foraging and shoaling.

The results of Experiment 1 demonstrate a fundamental form of numerical abilities, i.e., the ability to distinguish more from less. They appeared to show that item number is a salient feature that can drive choice in angelfish. However, the question remained whether angelfish relied upon the number of food items in the sets or upon non-numerical variables that covaried with the number. In Experiment 1, we did not control several continuous attributes of the stimuli that could have facilitated the differentiation between the sets. We only controlled the inter-item distance, keeping it the same across all contrasts, and thus did not examine the influence of this variable when it was varied between the numerically different sets. However, this is one of the non-numerical variables that has been shown to affect discrimination between food sets in angelfish (Gómez-Laplaza and Gerlai, 2020). In the latter study, angelfish showed preference for the clustered (denser) sets over the scattered sets when the number and size of the items in the contrasted sets were the same. This finding was consistent in both the small and the large number range. In Experiment 2, we tested the potential effect of this variable differently by conflicting item numbers and item density to examine which feature of the presented items takes priority for angelfish in their choice. Interestingly, the angelfish’s choice depended upon whether the contrasted sets belonged to the small (item number ≤4) or the large number range (item number >4). In the large number range, we found the density of the contrasting sets to drive the choice, with the clustered (higher density) items being preferred to the lower density (scattered) items. Whereas in the small number range, the conflict between item density and number resulted in no clear or significant pattern of choices, suggesting that perhaps item number and density played similar roles. The other behavioral parameters we measured were all in-line with the above findings. For example, in the small number range, no significant preference was found in any of the contrasts according to the frequency of entry, the latency of entry, and the number of fish whose first choice was one or the other set. However, in the large number range, angelfish entered the zone by the numerically smaller clustered set (five food items) significantly more frequently than the zone by the opposite side item set. The rest of the behavioral parameters also indicated a similar tendency, albeit non-significant. We also note that in all the contrasts, a tendency to initially prefer the numerically smaller clustered set was apparent as indicated by both the first choice made by most subjects and the shorter latency to enter the zone close to that set. These results suggest that angelfish attended initially to the clustered sets. A similar preference for the dense over the scattered food sets has been observed in a previous study with angelfish (Gómez-Laplaza and Gerlai, 2020). This density bias (i.e., the general preference for dense over scattered items) has also been reported in a variety of other animal species (Emmerton, 1998; Stevens et al., 2007; Uller et al., 2013; Parrish et al., 2017, 2020; Bertamini et al., 2018).

In social species, such as angelfish (Praetorius, 1932), that compete for food, it may be advantageous to choose the dense set over a scattered set. According to optimal foraging theory (Stephens and Krebs, 1986), in a situation in which fish are not food deprived and food is abundant enough, the most efficient strategy for a solitary forager would be to choose food items spaced closed to each other, because it would require less movement (minimizing detection risk) and thus less energy to collect o catch the food items. In addition to the energy expenditure associated with collecting food items, clustered food items are easier to monopolize than more scattered ones. Thus, territoriality and social interaction may also influence food choice in social species like the angelfish, a prediction that implies a complex interplay between the features of the food item sets contrasted and the social context.

A preference for dense stimuli has also been demonstrated in angelfish in a social context, i.e., when the choice offered was between two shoals (groups) of conspecifics. The effect of stimulus shoal density (the number of stimulus fish per volume of water) on angelfish choice was found to depend upon whether the contrasted stimulus shoals belonged to the small (Gómez-Laplaza and Gerlai, 2011b) or the large number range (Gómez-Laplaza and Gerlai, 2013). As in the current study, these prior studies demonstrated a significant effect of density in the large but not in the small number range. The density of the stimulus shoals was found to affect shoaling preferences also in other fish species such as the three-spined stickleback (Frommen et al., 2009).

However, not all species exhibit the above-described item-density dependent preference, and similarly, not all experimental procedures deliver this result (e.g., Rugani et al., 2008; Dadda et al., 2009; Agrillo et al., 2010). Thus, it appears that the influence of item density in quantity discrimination tasks is dependent upon the context, the procedure, the number range, and the species used.

In Experiment 3, we made both convex hull and cumulative contour length of the presented items constant between the contrasting sets to investigate the potential effects of other factors. Under these circumstances, we found angelfish to generally prefer the numerically smaller sets in which the items were larger-sized and presented more scattered. This preference was more robust in the large number range (10 vs. 5 items) but was also found for contrasts in the small number range. For example, in the 3 vs. 2 contrasts all parameters measured, except the preference index, indicated a significant preference for the smaller set, and a non-significant tendency to favor the smaller set was also found in the 4 vs. 2 contrasts in all behavioral parameters measured. Additionally, no significant difference between the three contrasts was found in the amount of time spent close to the numerically smaller sets. Overall, the results suggest that when the contrasted sets presented the same total amount of food (cumulative contour length) occupying the same space (convex hull), the size of the food items in the sets took priority over other features of the item sets, including the number of items and inter-item distance. We emphasize that these latter features were found important in previous studies that investigated angelfish choice between simultaneously presented item sets (Gómez-Laplaza et al., 2018; Gómez-Laplaza and Gerlai, 2020). Thus, we conclude that food item size is a highly salient feature for angelfish, a conclusion consistent with recent findings obtained in zebrafish that showed great accuracy in discriminating fine size differences in objects (Santacà et al., 2020).

Few studies on quantity discrimination in fish have demonstrated that the number of food items is not the only factor in choosing between contrasted food item sets. Both angelfish (Gómez-Laplaza et al., 2019) and guppies (Lucon-Xiccato et al., 2015) were found to prefer larger-sized food items as opposed to larger food amounts. This result also implies an interplay between energy expenditure and energy gain associated with foraging similar to what we suggested above, as catching a single larger-sized food item may require less energy than collecting several smaller sized food items. A similar optimization strategy, i.e., preference for the larger-sized single food item in a set, has also been found in various non-human primate species (Boysen et al., 2001; Stevens et al., 2007; Beran et al., 2008).

In this study, we decided to not specifically explore the effect of the convex hull on angelfish’s choice between contrasted food item sets because prior studies indicated the role of this non-numerical feature to be negligible or unimportant. For this reason, we kept this variable constant across the contrasts presented in Experiment 3. But convex hull was not dissociated from other, numerical and non-numerical, features of the contrasting sets in experiments 1 and 2. Although unlikely to have an important effect in the current study, we note that total amount of items presented, or the overall area occupied by the items may affect, for example, in the social context, i.e., when fish chose between two shoals (Agrillo et al., 2009; Gómez-Laplaza and Gerlai, 2013) as well as in other contexts and species (Feigenson et al., 2002; Agrillo et al., 2008; Vonk and Beran, 2012; Bogale et al., 2014; Gatto and Carlesso, 2019). Yet, in other studies and species, convex hull, i.e., the area occupied by the stimuli presented did not affect choice (Dadda et al., 2009; Lucon-Xiccato et al., 2018). Thus again, it appears that the effect of total item amount or the area occupied by these items depends upon, study-specific experimental procedure, context, and species.

The last question, we wish to consider concerns our results suggesting performance differences in angelfish choosing between food item sets in the small vs. in the large number range. Briefly, our results suggest that the continuous variables angelfish use in food quantity discrimination depend upon whether the contrasted food items/amounts are numerically large or small. A similar number range dependent effect has also been observed in the social context in angelfish (Gómez-Laplaza and Gerlai, 2011b, 2013), in other animal species (e.g., Rugani et al., 2011) including in other fish species (Agrillo et al., 2008, 2009, 2010) as well. Number range specificity is usually interpreted as proof for the study species using distinct representational mechanisms for their choice. For example, discrimination of small quantities is proposed to be based essentially upon a mechanism of visual attention and is termed an object file system (OFS). In this system, each element is stored in working memory as an individual item, the system can simultaneously represent only a limited number of items, which, for most species, is about 3–4 (Trick and Pylyshyn, 1994). For large numbers, however, a different mechanism, termed approximate number system (ANS), has been proposed. Using this latter mechanism, discrimination depends upon the ratio between the two contrasted quantities, and, unlike in OFS, not upon the absolute numerical difference between them. Hence, ANS adheres to Weber’s law: performance decreases as the numerical ratio approaches one (Feigenson et al., 2004). Some evidence also suggests that ANS may function in the entire numerical range, i.e., from small to large (Cantlon and Brannon, 2006; Pepperberg, 2012; Rugani et al., 2014). The results of Experiment 1 are consistent with the use of the ANS in angelfish. The performance of angelfish was similar when choosing between food sets of unequal sizes both in the large and in the small number range, including comparisons with the same ratio of 2:1 (i.e., 4 vs. 2 and 10 vs. 5). However, in Experiments 2 and 3, we found angelfish to perform distinctly depending on the number range to which the contrasts belonged. In the large number range, the presented contrast was discriminated against, but in the small number range, it was not. Since in angelfish four items exceed the upper limit of OFS-based discrimination (Gómez-Laplaza and Gerlai, 2011b; Gómez-Laplaza et al., 2018), the contrast 4 vs. 2 could be considered as crossing the boundary of the large/small set. Thus, the failure in the discrimination may be the result of having to use two distinct representational systems for the same contrast, as argued by Cordes and Brannon (2009), a conflict between representational systems that impedes comparison. However, our current experiments were not designed to systematically explore representational systems in angelfish and thus the above working hypothesis will need future empirical analysis.

In summary, our study demonstrates that angelfish do not choose according to one rigid rule. Their preference for one of the sets in a two choice task depends upon multiple factors. For example, not one of the features of the contrasting sets enjoys consistent priority. Instead, angelfish likely weighs the relative importance of multiple features of the contrasting sets to optimize its energy gain, i.e., to minimize energy expenditure and maximize energy intake. It is also notable that this optimization strategy likely depends upon the context, e.g., environmental circumstances, in which the choice presents itself. Thus, optimizing energy gain may have to be balanced against the risk of predation and social conflicts, i.e., interactions not just with the inanimate features of the environment but also with its animate characteristics, hypotheses whose validity we will explore in the future.

## Data Availability Statement

The datasets generated for this study are available on reasonable request to the corresponding author.

## Ethics Statement

The animal studies were reviewed and approved by the Ethics Committee of the University of Oviedo (permit ref.: 13-INV-2010).

## Author Contributions

LG-L conceived the study, elaborated the stimuli, collected the data, prepared the figures, analyzed the data and interpreted the results. LG-L and RG designed the study, wrote the article, and approved it for publication.

## Conflict of Interest

The authors declare that the research was conducted in the absence of any commercial or financial relationships that could be construed as a potential conflict of interest.
